# Self-assembly of ternary hollow microspheres with strong wideband microwave absorption and controllable microwave absorption properties

**DOI:** 10.1038/s41598-017-08293-3

**Published:** 2017-08-16

**Authors:** Qiang Zeng, Ping Chen, Qi Yu, Hai-rong Chu, Xu-hai Xiong, Dong-wei Xu, Qi Wang

**Affiliations:** 1 State Key Laboratory of Fine Chemicals, School of Chemical Engineering, Dalian University of Technology, Dalian, 116024 China; 2 Liaoning Key laboratory of advanced polymer matrix composites, Shenyang Aerospace University, Shenyang, 110136 Shenyang, China

## Abstract

In this study, we report a simple and efficient two-step method consisting of water-in-oil (W/O) emulsion technique and subsequent annealing process for synthesizing the hollow reduced graphene oxide microspheres embedded with Co nanoparticles (Air@rGO€Co). The microspheres showed good electromagnetic properties because of the coexistence of magnetic loss and dielectric loss to microwaves. The minimum reflection loss (RL_min_) value of S_1.5_ reaches −68.1 dB at 13.8 GHz with a thickness of 2.2 mm, and the absorption bandwidth (lower than −10 dB) is 7.1 GHz covering from 10.9 GHz to 18.0 GHz. More interestingly, we can easily controll the microwave absorbing properties of the microspheres by changing the ratio of the two components in the composites. The excellent electromagnetic match at the corresponding resonance peaks for dielectric and magnetic loss play an important role in improving microwave absorption property. Our study provides a good potential method for preparation of lightweight microwave absorbing materials.

## Introduction

The rapid development of modern science and technology such as radar technology and modern electronic information industry has promoted increasing demand for high effective microwave absorbing (MA) materials. For example, stealth airplane, stealthy warship, and microwave darkroom^1–3^. The ideal microwave absorbing materials should possess the characteristics of thin thickness, low density, strong absorption over a broad frequency, and good chemical stability^4, 5^. Generally, basis on their microwave loss mechanism, the MA materials can be classified into two categories, including dielectric loss and magnetic loss^6^. The loss mechanism of dielectric materials such as carbon nanotubes (CNTs), graphene, carbon nanofibers (CNFs) and reduced graphene oxide (rGO) is mainly attributed to their dipolar relaxation polarization. These carbon-based materials although have some advantages such as chemical stability and light weight, but the out of balance of their dielectric permittivity and magnetic permeability will lead to most of the incident microwaves being reflected rather than absorbed^5, 7^. While the magnetic materials such as Fe_3_O_4_, Co_3_O_4_, Fe, Ni and Co although have strong magnetic loss, but the problems such as easy oxidation and high density have also hampered their applications^8^.

Thus, constructing the composite absorbers with both high dielectric and magnetic loss has attracted a lot of attention, which always exhibit strong microwave absorption properties. Wang and their colleagues prepared graphene@Fe_3_O_4_@C@PANI composites with the mimimum reflection loss (RL) of the composites reached −44.2 dB at 11.4 GHz at the thickness of 3.0 mm^9^. Li *et al*. prepared SWCNT/CoFe_2_O_4_ nanocomposites with the mimimum reflection loss at 12.9 GHz and the reflection loss value of −30.7 dB at a thickness of 2.0 mm^10^. Wang *et al*. reported excellent MA material *via* an *in situ* oxidation polymerization of pyrrole in an aqueous dispersion of Co nanoparticles. The as-synthesized composites with a coating layer thickness of 2.0 mm exhibited a maximum absorption of −33dB at 13.6 GHz as well as a bandwidth of 4.8 GHz (at a frequency of 11.7–16.5 GHz) corresponding to reflection loss lower than −10 dB^11^. Zong *et al*. synthesized RGO/CoFe_2_O_4_ composites by a one-pot hydrothermal route^12^, the mimimum RL of the composite is −47.9 dB at 12.4 GHz with a thickness of 2.3 mm, and from 12.4 to 17.4 GHz the RL of the composites below −10 dB. Huang *et al*. synthesized graphene@Fe_3_O_4_@carbon nanocomposites, the mimimum reflection loss of the nanocomposites is −30.1 dB at 14.8 GHz with a thickness of only 1.8 mm, and the reflection loss below −10dB is ranges from 12.1 to 17.5 GHz^13^. Ren *et al*. prepared RGO–CoFe_2_O_4_/GNSs composites and obtained the minimum reflection loss value of −21.8 dB at 11.8 GHz with 13 wt% filler content, and the frequency bandwidth less than −10 dB is from 9.6 to 12.4 GHz^14^. Pan *et al*. studied the microwave absorption properties of α-Co/graphene composites and β-Co/graphene composites, the results showed that the α-Co/graphene composites have excellent MA property, and the matching frequency for reflection loss exceeded −47.5 dB was at 11.9 GHz^15^.

In the last few years, microcapsule techniques have gained an increasing attention due to their high potentialities in many fields, such as self-healing materials, drug delivery and anti-corrosion field. White *et al*. prepared a structural polymeric material with the ability to autonomically heal cracks^16^. The material incorporates a microencapsulated healing agent that is released upon crack intrusion, afte that, polymerization of the healing agent is then triggered by contact with an embedded catalyst, and bonding the crack faces. The fracture experiments yield as much as 75% recovery in toughness. Xiong *et al*. report a novel capsule-based selfrecovery system that utilizes chloride ions as a trigger^17^. Those capsules, which are functionalized via a smart response to chloride ions, are fabricated using a silver alginate hydrogel that disintegrates upon contact with chloride ions, and thereby releases the activated core materials which can be precipitated with chloride ions. They introduce the capsules into cementitious materials, and prove the capsules still can be responsive to chloride ions in concrete matrix. Therefore, the capsules will be a promising candidate for building materials and prolong the life of construction and building materials, especially in marine environment. Wang *et al*. fabricated a self-immunity microcapsule which can be triggered by low pH values, and calcium hydroxide can be controllably released to regulate the environmental pH condition and decrease the [Cl^−^]/[OH^−^] ratio, reach corrosion protection of steel bar in reinforced concrete. Furthermore, test results show that the release rate of core materials could interact with environmental pH value; the rate increases markedly with decreasing pH value, but is inhibited by high pH values^18^. Cui *et al*. fabricated multi-stimuli responsive smart chitosan-based microcapsules (MSRS-CS-MCs) via a facile sonochemical method^19^. The obtained spherical MSRS-CS-MCs with the average size of 500 nm showed excellent magnetic responsive ability, favorable selectively folate-receptor-mediated targeting functionality to the HeLa cells, and attractive reduction-responsive release ability for hydrophobic drugs, which make MSRS-CS-MCs promising nanocarriers for future biomedical applications.

MA performances of microwave absorbing materials are closely related to their microstructures^20, 21^. In order to improve the dielectric-magnetic composite MA performance further, many efforts have been devoted to developing novel MA absorber with special structures, such as barium ferrite@poly (3,4-ethylenedioxy thiophene), Fe_3_O_4_@TiO_2_, Fe_3_O_4_@ZnO, Ni@P, CoNi@SiO_2_@TiO_2_, CoNi@Air@TiO_2_^1, 22–27^. These core/shell or Yolk/shell multicomponent hierarchical microspheres with magnetic/dielectric absorption components, can generate tunable reflection loss by changing the type and relative content of core/shell component, thereby improving the impedance matching in terms of complex permittivity and permeability. Typically, based on our previous works, it confirms that hollow hybrid 3D microspheres have excellent MA performance^28^.

Herein, we report a facile and efficient procedure for preparation of hollow hybrid 3D microspheres (Air@rGO€Co microspheres), in which Co nanoparticles were embedded within the spongy shell assembled by rGO nanosheets. These Air@rGO€Co microspheres show strong wide band and controllable microwave absorption properties. The minimum reflection loss (RL_min_) value of S_1.5_ reaches −68.1 dB at 13.8 GHz with a thickness of 2.2 mm, and the absorption bandwidth (lower than −10 dB) is 7.1 GHz covering from 10.9 GHz to 18.0 GHz. The excellent absorption capability could be attributed to the special 3D morphology with hollow cavities, the intrinsic magnetic loss from Co nanoparticles and dielectric polarization loss from the rGO nanosheets. Interestingly, we can easily controll the microwave absorbing properties of the microspheres by changing the ratio of the two components in the composites.

## Experimental section

### Materials

All chemical regents including concentrated hydrochloric acid (HCl), polyvinyl alcohol, ethyl alcohol and acetylacetone cobalt(II)(AACo) were purchased from Sinopharm Chemical Reagent Co., Ltd (Shanghai, China). Olive oil (Olivoilà) and deionized water obtained from a Milli-Q system (Millipore, Bedford, MA) were used.

### Preparation of GO aqueous dispersion

GO was synthesized from natural graphite powder using a modified Hummers method^28^. The resulting solid GO were dispersed in water under ultrasound treatment to obtain an aqueous dispersion of GO nanosheets (4.5 mg ml^−1^).

### Synthesis of the Air@rGO€Co microsphere

**Figure 1:**
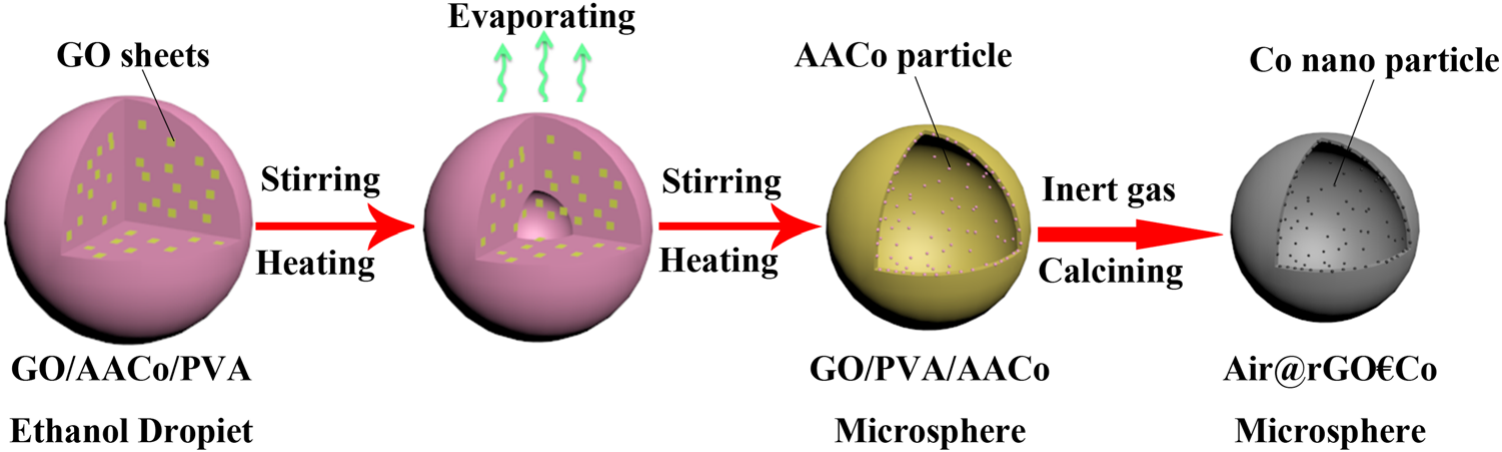
A schematic illustration for the self-assembly process of Air@rGO€Co microspheres.

Polyvinyl alcohol (PVA, 0.3 g), acetylacetone cobalt (AACo, 1.5 g) and absolute ethyl alcohol (50 ml) were mixed with GO suspension (4.5 mg ml^−1^, 20 ml) and stirred for several hours to obtain homogeneous suspension. The obtained suspension was then slowly added into the hot olive oil (preheated to 75 °C) under intense stirring (6000 r min^−1^) to form stable water-in-oil emulsions. The emulsion system was kept stirring with speed of ~500 r min^−1^ for two hours at 75 °C to remove the ethyl alcohol, followed by heated to 95 °C with continuously stirring for another two hours to remove the water and the remaining ethyl alcohol. Finally, the emulsion system was cooled down to room temperature and the GO/AACo/PVA microspheres were separated and purified by centrifugation, washing and drying. The obtained microspheres precursor was calcined at 550 °C in Ar atmosphere for two hours to obtain the Air@rGO€Co microspheres and labeled as S_1.5_. The schematic illustration for the self-assembly process of Air@rGO€Co microspheres is shown in Fig. 1. For comparison, GO/AACo/PVA microspheres with different amounts of AACo (0, 0.5, 1.0, 2.0, and 2.5 g) were addedthe, and the corresponding Air@rGO€Co microspheres were labeled as S_0_, S_0.5_, S_1.0_, S_2.0_ and S_2.5_, respectively. Pure Co nanoparticles were prepared by calcining AACo in the same conditions and labeled as S_p_.

### Characterization

The composition and phase purity of the as-synthesized samples were analyzed by X-ray diffraction (XRD) with monochromatized Cu-Kα incident radiation by a Rigaku D/Max-2400 instrument operating at 12 kV voltage. The size and morphology of the samples were characterized by a Hitachi Su3500 scanning electron microscope (SEM) equipped with an energy dispersive spectrometer (XFlash 5030, Bruker, Germany), along with a field–emission transmission electron microscope (TEM, Tecnai F30, USA). AFM analysis was conducted on a PicoScanTM 2500(USA). X-ray photoelectron spectroscopy (XPS) was recorded on an ESCALAB 250 spectrometer (Thermo Fisher) to characterize the surface composition. The complex permittivity and permeability were measured using the waveguide technique at the frequency range of 1−18 GHz with an Agilent 8720ET network analyzer. These products were uniformly blended with paraffin matrix with a mass ratio of 1:2, and the mixture was cast into a ring mold with thickness of 2.0 mm, inner diameter of 3 mm, and outer diameter of 7 mm.

## Results and Discussion

**Figure 2:**
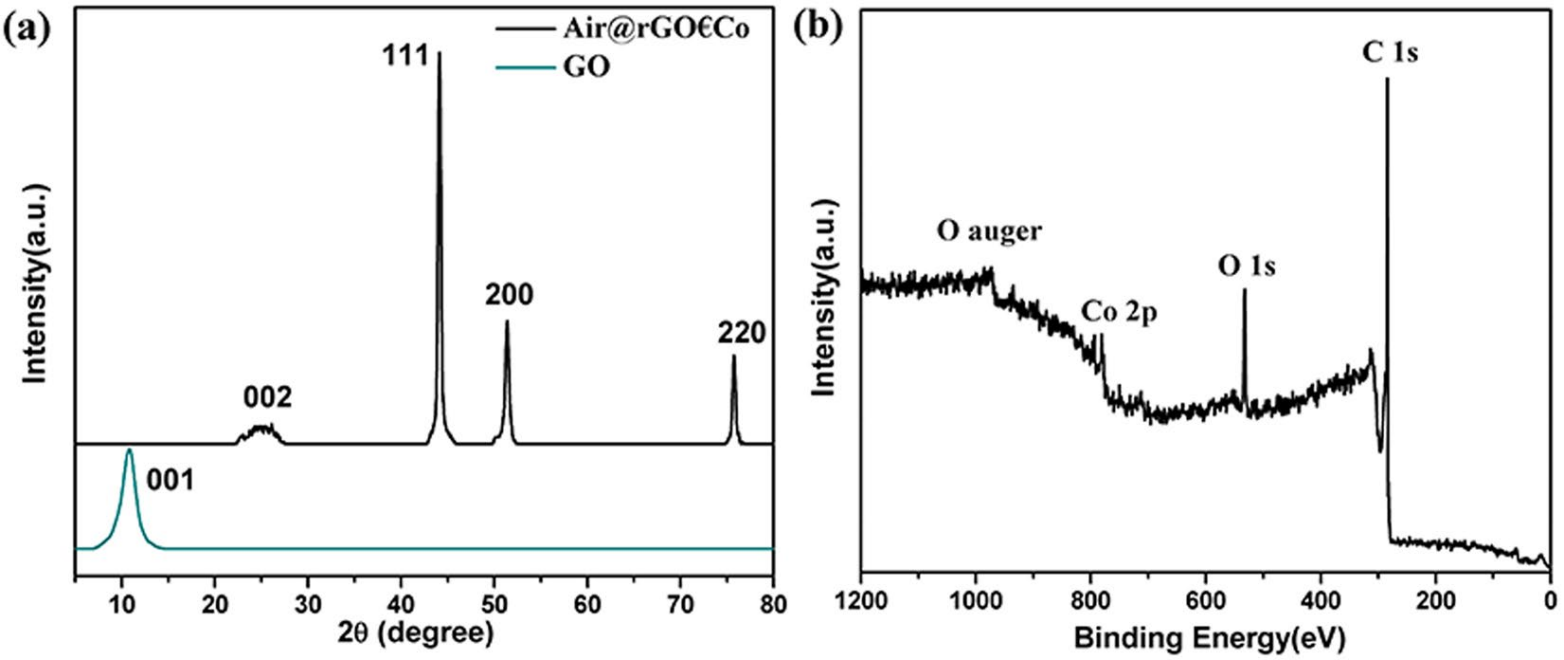
(**a**) XRD patterns of S_1.5_ (**b**) The XPS full spectrun of S_1.5_.

The synthesis process of Air@rGO€Co microsphere is depicted in Fig. 1, where PVA acts as the binder between AACo particles and GO nanosheets as well as the framework of microsphere, which can be ablated off after calcination in Ar atmosphere. In the calcination process, GO nanosheets which have been reduced to rGO were linked together by the reactions of rested hydroxy, epoxy and carboxy group. Simultaneously, PVA was pyrolyzed and AACo particles were calcined to be Co nanoparticles at high temperature. The crystallinity of the nanoparticles was characterized by X-ray diffraction (XRD). As seen from Fig. 2a, all diffraction peaks can be assigned to the (111), (200) and (220) lattice planes of the cubic spinel structured Co (JCPDS No.15–0806). The appearance of diffraction hump (002), which originates from the short-range order of stacked graphene sheets, and the disappearance of diffraction peak (001) confirm that the GO sheets were completely reduced in the calcination process^29, 30^. The chemical composition of S_1.5_ was studied by X-ray photoelectron spectroscopy (XPS). The peaks associated with C 1 s, O 1 s, and Co2p indicate that the microsphere consists of three elements including C, O and Co (Fig. 2b).

**Figure 3:**
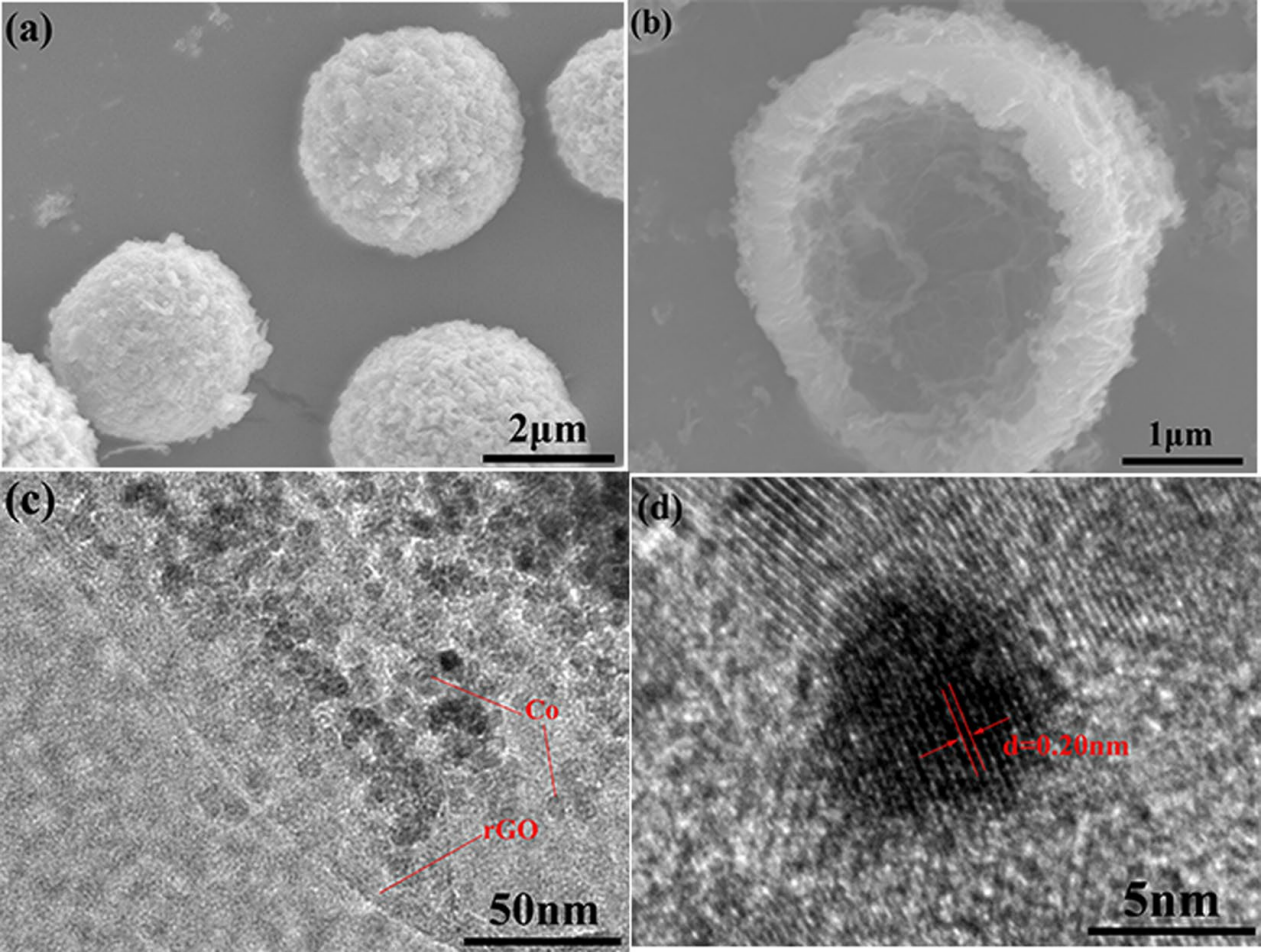
SEM images of (**a**) Air@rGO€Co microspheres (S_1.5_), (**b**) cross-section of microsphere. (**c**) TEM image of a fragment from a microsphere which has been ultrasonic decomposed, (**d**) high magnification TEM image of the fragment.

Figure 3a,b show a representative scanning electron microscopy (SEM) images of the Air@rGO€Co microspheres (S_1.5_), which show hollow spherical structure. The size of microspheres is not uniform and their diameters are normally distributed, and the size distribution analysis of Air@rGO€Co microspheres was shown in Fig. S1 (Supplementary information). The result shows that the range of microspheres diameters is 3 to 6 μm, and the mean diameter is 4.2 μm. There presents a hollow-core construction in the interior of the microspheres and the thickness of the spongy shell is about 0.5 μm (Fig. 3b). Transmission electron microscopy (TEM) images in Fig. 3c show that Co nanoparticles are sandwiched between the rGO nanosheets with an average size of 7 nm. Moreover, the high-resolution image (as shown in Fig. 3d) revealed a distance between atoms of *ca*. 0.20 nm, which corresponds to the distance between the (111) planes of Co. The energy disperse spectroscopy (EDS) elemental mappings (Fig. S2d–f, Supplementary information) show different distribution patterns for C, O and Co elements. The EDS line scanning analyses (Fig. S2c, Supplementary information) reveal that the relative concentration of Co element increases from outer to inner surface of the microsphere wall, which will be benefit to improve the corrosion resistance of the Air@rGO€Co microspheres.

The electromagnetic properties (complex permittivity and permeability) of Air@rGO€Co microspheres were investigated in the frequency range of 1.0−18.0 GHz at room temperature. The real permittivity (*ε′*) and real permeability (*μ′*) represent the storage ability for electromagnetic energy, and the imaginary permittivity (*ε″*) and imaginary permeability (*μ″*) are an expression for the dissipation of energy and magnetic loss, respectively^31^. The dielectric loss tangent (*tan δε = ε″/ε′*) and the magnetic loss tangent (*tan δμ = μ″/μ′*) are used to describe the corresponding dielectric and magnetic loss ability^32, 33^.

**Figure 4:**
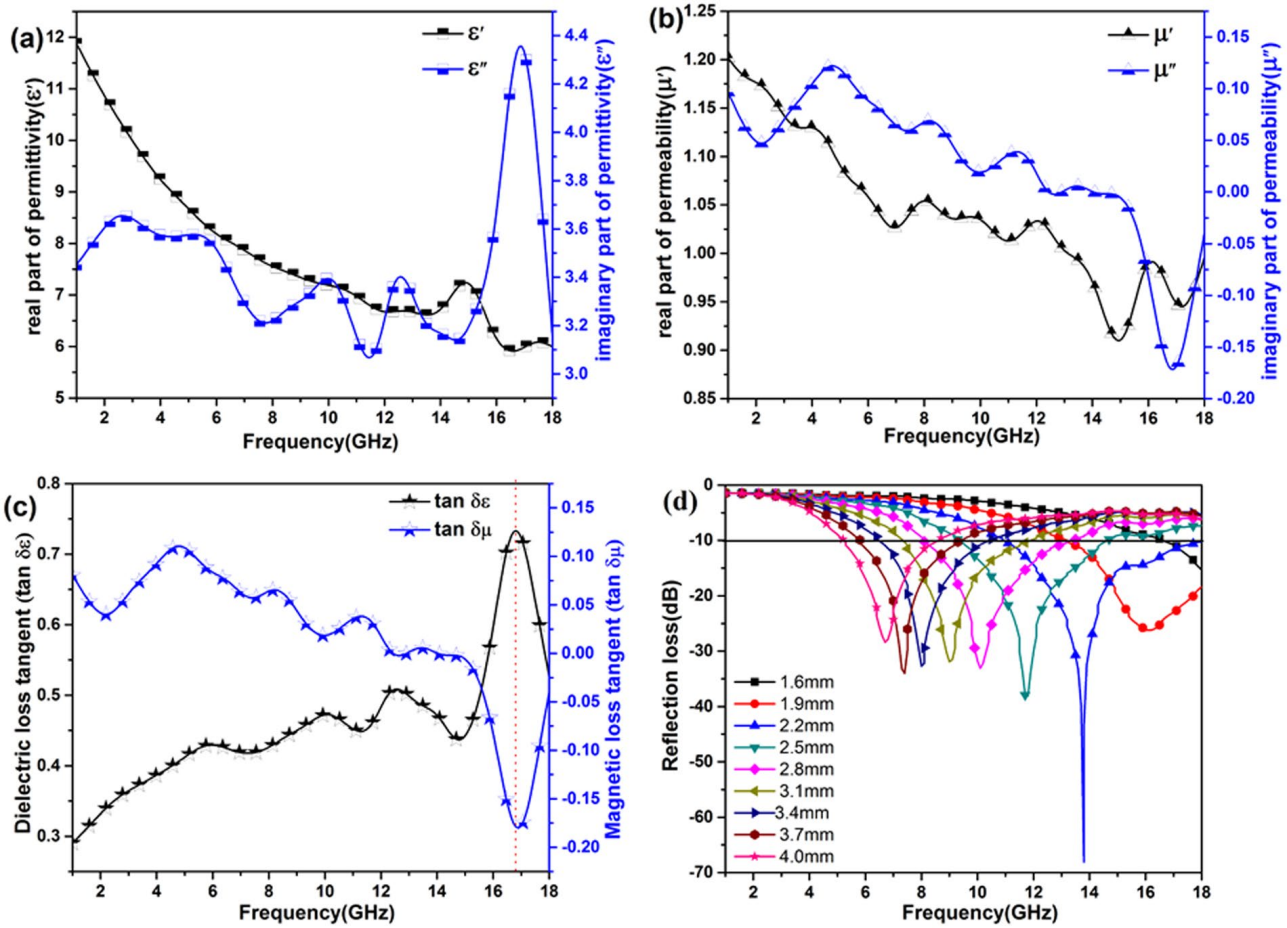
Relative complex permittivity (**a**), relative complex permeability (**b**), dielectric loss tangent (tan *δ*_ε_) and magnetic loss tangent (tan *δ*_*μ*_) (**c**) of paraffin composites filled with 33.3 wt% S_1.5_, and the calculated reflection loss of paraffin composites with different thicknesses (**d**).

The samples used for the measurement of electromagnetic properties were prepared by mixing the paraffin wax and Air@rGO€Co microspheres (mass ratio was 2:1). As shown in Fig. 4a, the real part of permittivity (*ε′*) value of S_1.5_ is located within the range of 6–12, and the imaginary part of permittivity (*ε″*) value is located within the range of 3.1–4.3. The parameters of the relative complex permeability for the sample is shown in Fig. 4b, the real part of permeability (*μ′*) values display a decrease trend with the increase of frequency, and the imaginary part of permeability (*μ″*) value is within the range of 0.15–−0.17.

The dielectric loss tangent and the magnetic loss tangent are shown in Fig. 4c. The presence of multiple loss peaks on the dielectric loss tangent curve suggests complex dissipation mechanisms of dielectric loss process. Multiple polarization processes may cause these resonance peaks, including the interface polarization induced by the presence interface between the Co and rGO^34^, and the polarization of existing defects and functional groups in the rGO^35^. Multi-resonance peaks which are induced by multiple magnetic loss mechanisms can be observed at 2–16.5 GHz in the magnetic loss tangent curves^36, 37^. The peaks detected at the low-frequency range of 2–6 GHz are related to natural resonance^38^, and the resonance peaks appearing in the middle-frequency (6–12 GHz) are ascribed to the dipolar polarization and exchange resonances^36, 39, 40^. According to the Maxwell equations, a magnetic field can be induced by an *ac* electric field and the magnetic energy can transferred into the electric energy^41^. Thus, the magnetic loss tangent and increased dielectric loss tangent value of Air@rGO€Co microspheres in the frequency range of 15.0–18.0 GHz (Fig. 4c) denotes that the magnetic energy is transferred into the electric energy.

Based on above EM parameters, the reflection loss (RL) was calculated based on the relative complex permittivity and permeability at a given frequency and thickness according to the following equations^3, 38^

1 $${Z}_{in}=\sqrt{{\mu }_{r}/{\varepsilon }_{r}}tanh[-j(2\pi fd/c)\sqrt{{\mu }_{r}/{\varepsilon }_{r}}]$$

2 $$RL(dB)=20{\mathrm{log}}_{10}|({Z}_{in}-1)/({Z}_{in}+1)$$

where *ε*_*r*_ and *μ*_*r*_ are the relative complex permittivity (*ε*_*r*_ *= ε′ − jε″*) and permeability (*μ*_r_ = *μ*′ −*jμ*″) of the absorber, *f* is the frequency of microwave in free space, *c* is the velocity of light, *d* is the coating thickness and *Z*_in_ is the input impedance of the absorber. When the RL valve is lower than −10 dB, it means that only 10% of the incident microwave is reflected while 90% of the microwave is absorbed. Conventionally, we define the frequency range over which the RL value lower than −10 dB as the effective absorption bandwidth. Figure 4d shows the calculated RL of paraffin/S_1.5_ (mass ratio was 2:1) composites at different thicknesses (1.6–4.0 mm). The minimum reflection loss (RL_min_) value of the composites reaches −68.1 dB at 13.8 GHz (matching thickness is 2.2 mm), and the absorption bandwidth (lower than −10 dB) is 7.1 GHz covering from 10.9 GHz to 18.0 GHz. Otherwise, Fig. 4d presents an interesting phenomenon, as the matching thickness increased the reflection loss peak moved to a lower frequency region. The 1/4 wavelength equation can be used to explain above mentioned law^42^

3 $${t}_{m}={\rm{nc}}/4{{\rm{f}}}_{{\rm{m}}}{({\mu }_{r}{{\rm{\varepsilon }}}_{{\rm{r}}})}^{1/2}$$

where t_m_ is matching thickness, *f*_m_ is the frequency of the RL_min_ peaks, *ε*_*r*_ and *μ*_*r*_ are the complex permittivity and permeability, respectively, at the *f*_m_, and *c* is the velocity of light.

**Figure 5:**
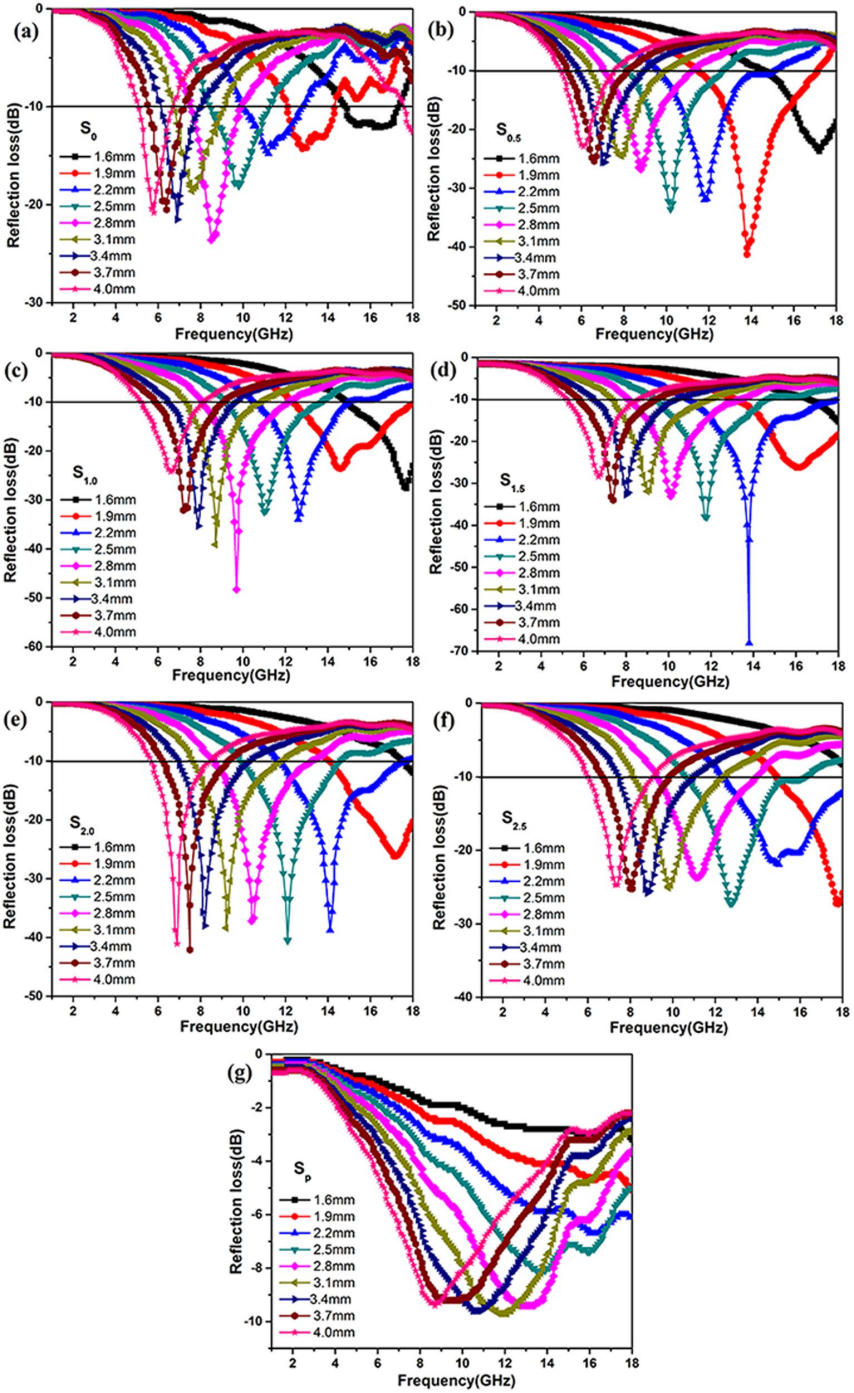
Calculated reflection loss of (**a**) S_0_, (**b**) S_0.5_, (**c**) S_1.0_, (**d**) S_1.5_, (**e**) S_2.0_, (**f**) S_2.5_, (**g**) S_p_ with differents thickness in the frequency range of 1–18 GHz.

Electromagnetic parameter is the key factor in improving the absorbing performance of the composites. Changing the ratio between AACo and the GO will obtained Air@rGO€Co microspheres with different electromagnetic parameter. In this way, the adjustable MA property of the composites can be realized. The electromagnetic parameters of S_0_, S_0.5_, S_1.0_, S_2.0_, S_2.5_ and S_P_ were shown in Figs S3–S8 (Supplementary information), and the reflection losses were calculated based on the electromagnetic parameters. As indicated in Fig. 5b–f, the Air@rGO€Co microspheres with different Co contents were also exhibit excellent microwave absorption ability. Comparing with the sample shown in Fig. 5a–g, it can be found that as the AACo content increase, the RL_min_ valve of Air@rGO€Co microspheres move to higher frequency range. The minimum reflection loss value (RL_min_), the frequency of RL_min_ value and the absorption bandwidth of differents samples under the thickness of 2.2 mm were shown in Table 1.

**Table 1:** The minimum reflection loss value (RL_min_), the frequency of RL_min_ and the absorption bandwidth of differents samples under the thickness of 2.2 mm.

Samples	RL_min_ [dB]	The frequency of RL_min_ [GHz]	Frequency range [GHz] (RL ≤ −10 dB)
S_0_	−14.8	11.2	10.0–12.7
S_0.5_	−32	11.8	9.7–15.1
S_1.0_	−34	12.6	10.6–14.9
S_1.5_	−68.1	13.8	10.9–18.0
S_2.0_	−38.1	14.1	11.7–17.7
S_2.5_	−21.1	15.0	12.4–18.0
S_p_	−6.7	16.4	—

The increasing content of AACo will introduce more magnetic component of Co nanoparticles into the microspheres. As shown in Figs S3–S8 (Supplementary information), the permeability of Air@rGO€Co microspheres increase with the increasing Co contents, thus results in the enhanced magnetic loss. The primary magnetic loss mechanism of Co nanoparticles is exchange resonance in higher frequency range^11^, so the microsphere with more Co content has a better absorption of microwave in higher frequency range. Meanwhile, the increasing content of Co means the density of rGO in the microspheres was decreased. On the contrary, the permittivity of Air@rGO€Co microspheres decrease with the increasing Co contents (Figs S3–S8, Supplementary information), meaning that the dielectric loss was weakened. Debye dipolar relaxation is main dielectric loss mechanism of rGO in lower frequency domain^28^, so the microsphere with more rGO content has a better absorption of microwave in lower frequency range.

As a result, we can easily controll the microwave absorbing frequency range and RL valve of the microspheres by changing the ratio of the two components in the Air@rGO€Co microspheres.

**Figure 6:**
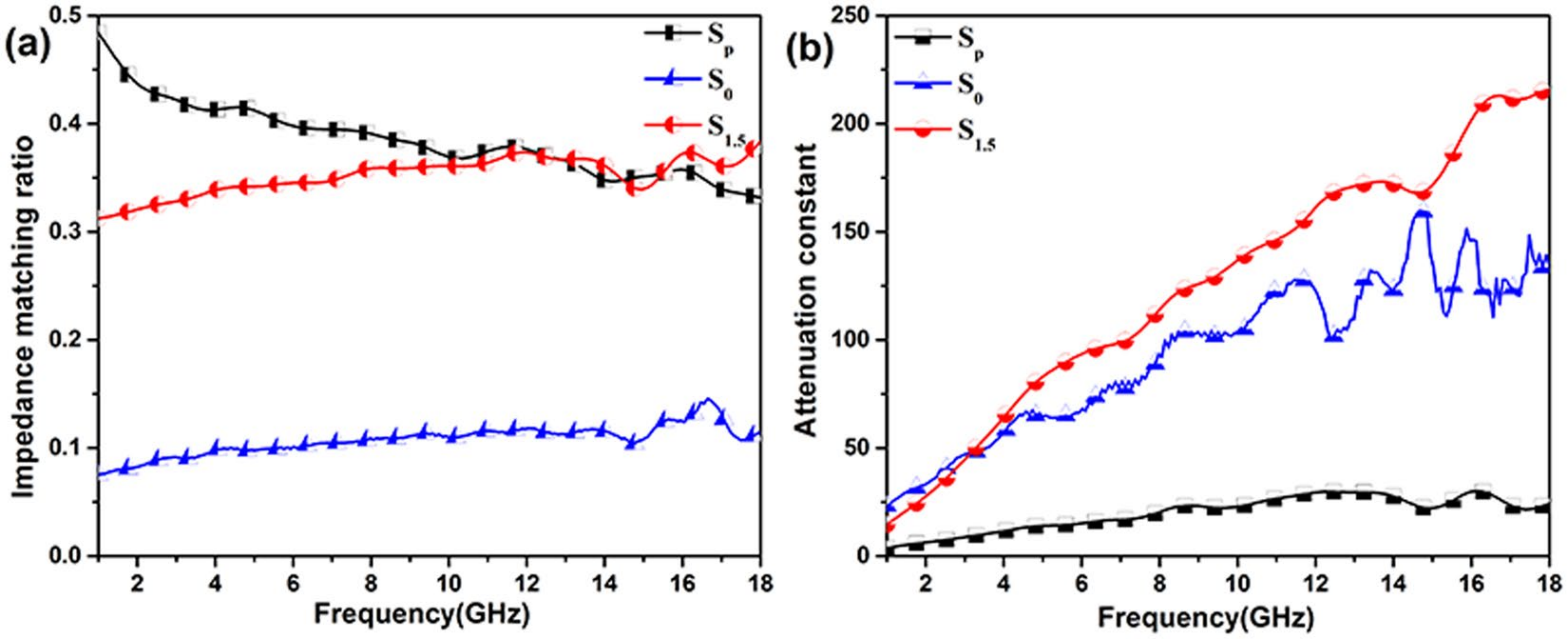
(**a**) Impedance matching ratio *Z*, (**b**) attenuation constant α of S_p_, S_0_ and S_1.5_.

From Fig. 5a,d and g, the minimum reflection loss value of S_0_ and S_p_ was no more than −25 dB, while that of S_1.5_ at a coating thickness of 2.2 mm was as much as -68.1 dB. The Air@rGO€Co microspheres which composed of dielectric loss material (rGO) and magnetic loss material (Co) exhibit strong microwave absorption properties. To evaluate the MA performance of material, impedance matching *Z* and attenuation constant α are two of the most important parameters, as expressed by the following equation^43^

4 $$Z=\sqrt{\sqrt{({\mu }^{^{\prime} 2}+\mu {^{\prime\prime} }^{2})}/\sqrt{(\varepsilon {^{\prime} }^{1}+\varepsilon {^{\prime\prime} }^{2})}}$$

5 $$\alpha =\frac{\sqrt{2}\pi f}{c}\times \sqrt{(\mu ^{\prime\prime} \varepsilon ^{\prime\prime} -\mu ^{\prime} \varepsilon ^{\prime} )+\sqrt{{(\mu ^{\prime\prime} \varepsilon ^{\prime\prime} -\mu ^{\prime} \varepsilon ^{\prime} )}^{2}+{(\mu ^{\prime} \varepsilon ^{\prime\prime} -\mu ^{\prime\prime} \varepsilon ^{\prime} )}^{2}}}$$

where *f* is the frequency of the microwave and *c* is the velocity of light. Good impedance matching behavior of an absorbent means that most of incident electromagnetic wave can enter into material interior and be attenuated, rather than being reflected on the material surface. It is observed from Fig. 6a that the S_0_ have the lowest impedance matching ratio value, while the S_p_ have the highest impedance matching ratio value but the lowest attenuation constant α (Fig. 6b). As a consequence, both the S_0_ and the S_p_ show relatively poor microwave absorption properties. In contrast, the S_1.5_ have a moderate impedance matching ratiowhich is lower than that of S_p_ and higher than that of S_0_ (Fig. 6a), and fascinating attenuation ability according to Fig. 6b. Their attenuation values are much higher than that of the S_p_ and the S_0_ over 1–18 GHz. Overall, the S_1.5_ which could effectively offset the drawbacks of the sole Co nanoparticles or rGO nanosheets, possess moderate impedance matching and splendid attenuation constant as well as the optimal MA properties.

## Conclusions

Summary, we have successfully synthesized Air@rGO€Co microspheres by a simple and efficient two-step method consisting of water-in-oil (W/O) emulsion technique and subsequent annealing process. The microspheres showed good electromagnetic properties because of the coexistence of magnetic loss and dielectric loss to microwaves. The minimum reflection loss (RL_min_) value of S_1.5_ reaches −68.1 dB at 13.8 GHz with a thickness of 2.2 mm, and the absorption bandwidth (lower than −10 dB) is 7.1 GHz covering from 10.9 GHz to 18.0 GHz. More interestingly, we can easily controll the microwave absorbing properties of the microspheres by changing the ratio of the two components in the composites. As the Co content increasing, the RL_min_ valve of Air@rGO€Co microspheres move to higher frequency rang. Our study provides a good potential method for preparation of lightweight microwave absorbing materials.

## Electronic supplementary material

Supplementary information
