# Supplementary material for: Self-assembly of ternary hollow microspheres with strong wideband microwave absorption and controllable microwave absorption properties
Source: Sci Rep. 2017 Aug 16;7:8388. doi: 10.1038/s41598-017-08293-3 (PMC5559606; doi:10.1038/s41598-017-08293-3)
Supplement: Supplementary file 1 — Supplementary information [file 41598_2017_8293_MOESM1_ESM.doc]

**Self-assembly of ternary hollow microspheres with** **strong wideband microwave absorption and** **controllable microwave absorption properties**

Qiang Zeng,^a^ Ping Chen,*^a^ Qi Yu,*^b^ Hai-rong Chu^a^, Xu-hai Xiong,^b^ Dong-wei Xu,^a^ Qi Wang,^b^

^a^State Key Laboratory of Fine Chemicals, School of Chemical Engineering, Dalian University of Technology, Dalian (116024), China.

E-Mail: chenping_898@126.com

^b^Liaoning Key laboratory of advanced polymer matrix composites, Shenyang Aerospace University, Shenyang (110136), China

E-Mail: yuqi1027@126.com; chenping_898@126.com

**Supplementary information**


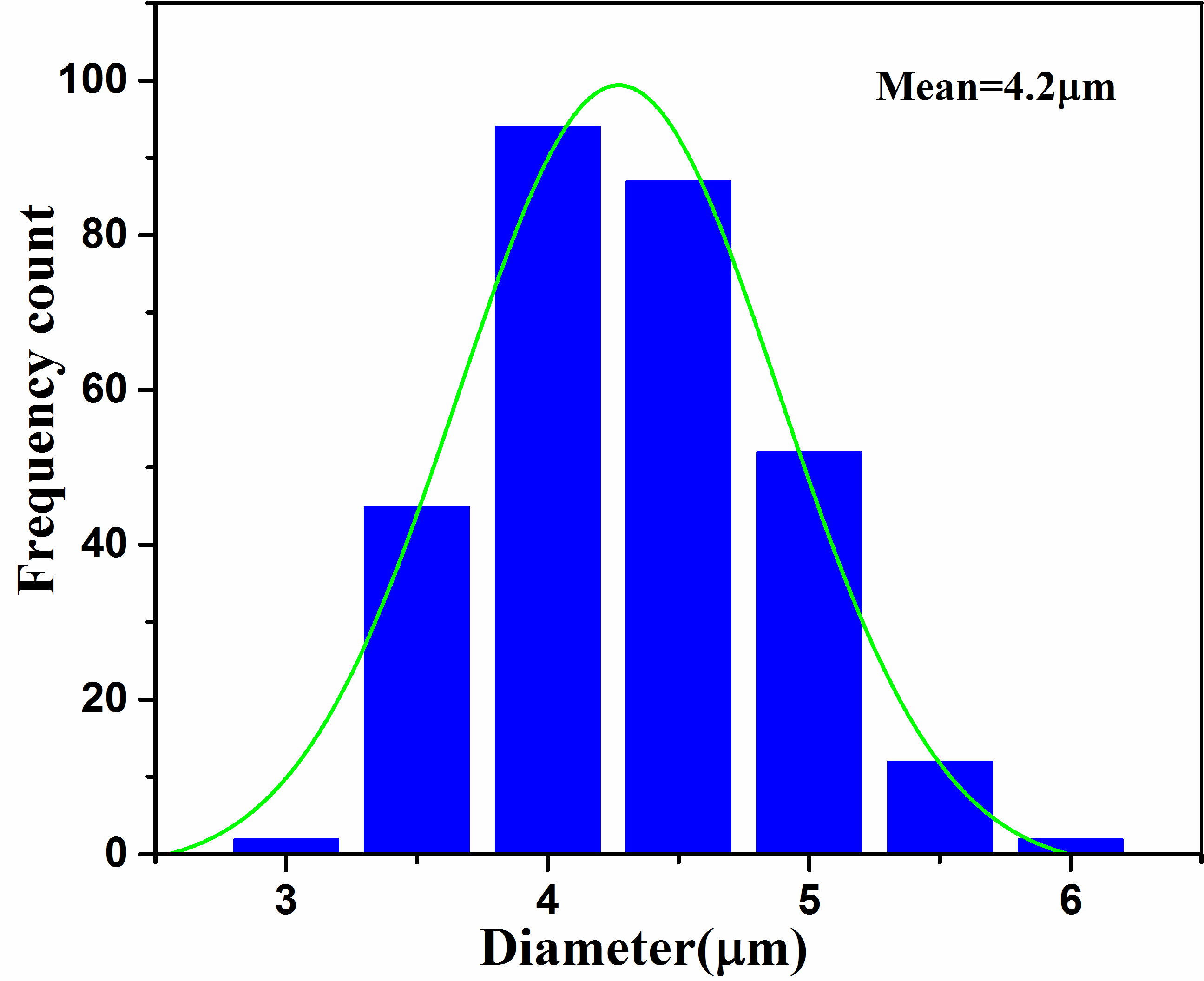


Fig.S1 The size distribution analysis of Air@rGO€Co microspheres

**
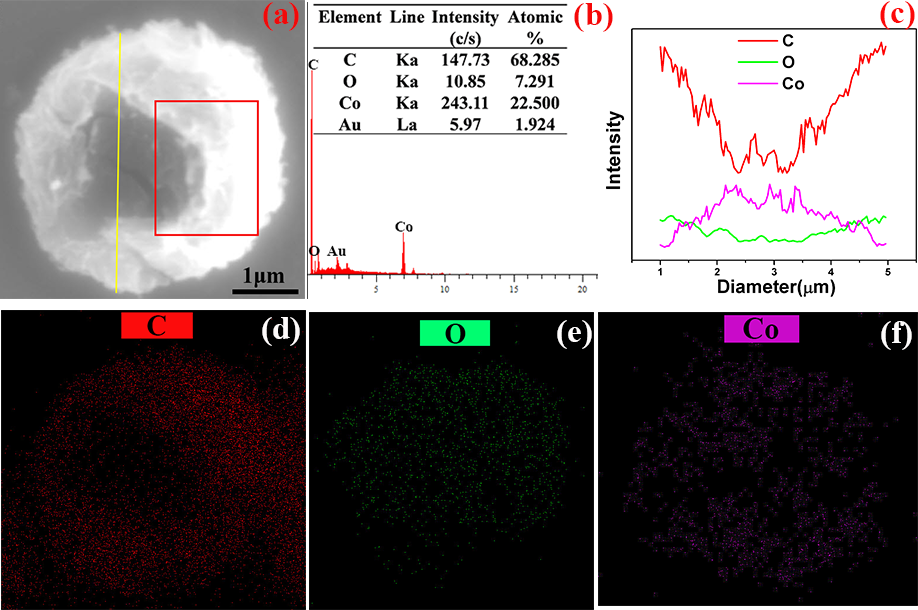
**

Fig.S2 (a) The cross-section of Air@rGO€Co microsphere; (b) EDS spectrum of the red area shown in panel (a); (c) Line scanning profiles of different elements recorded along the yellow line shown in panel (a); (d-f) EDS elemental mapping images of (d) C, (e) O and (f) Co obtained from (a) a single Air@rGO€Co microsphere.


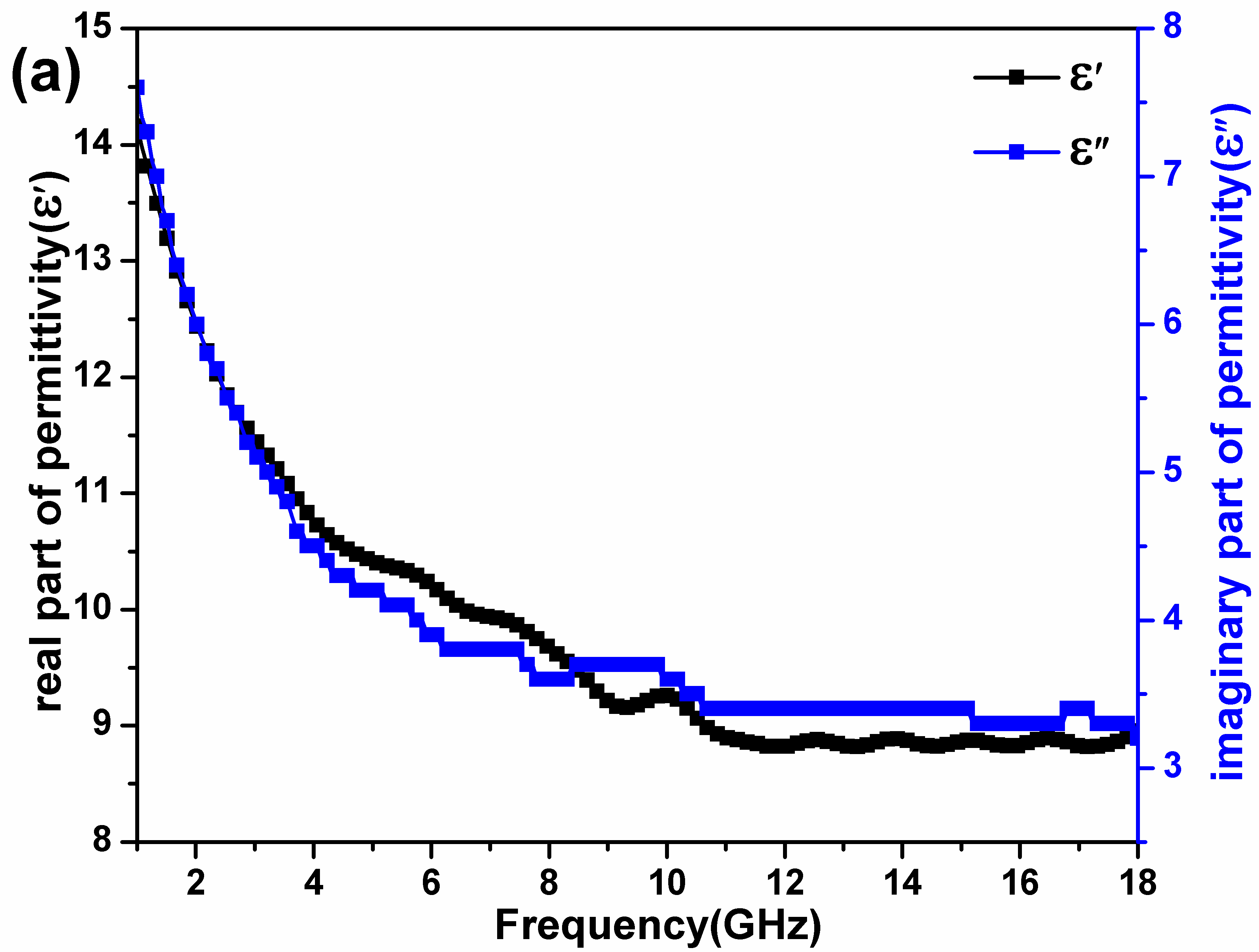

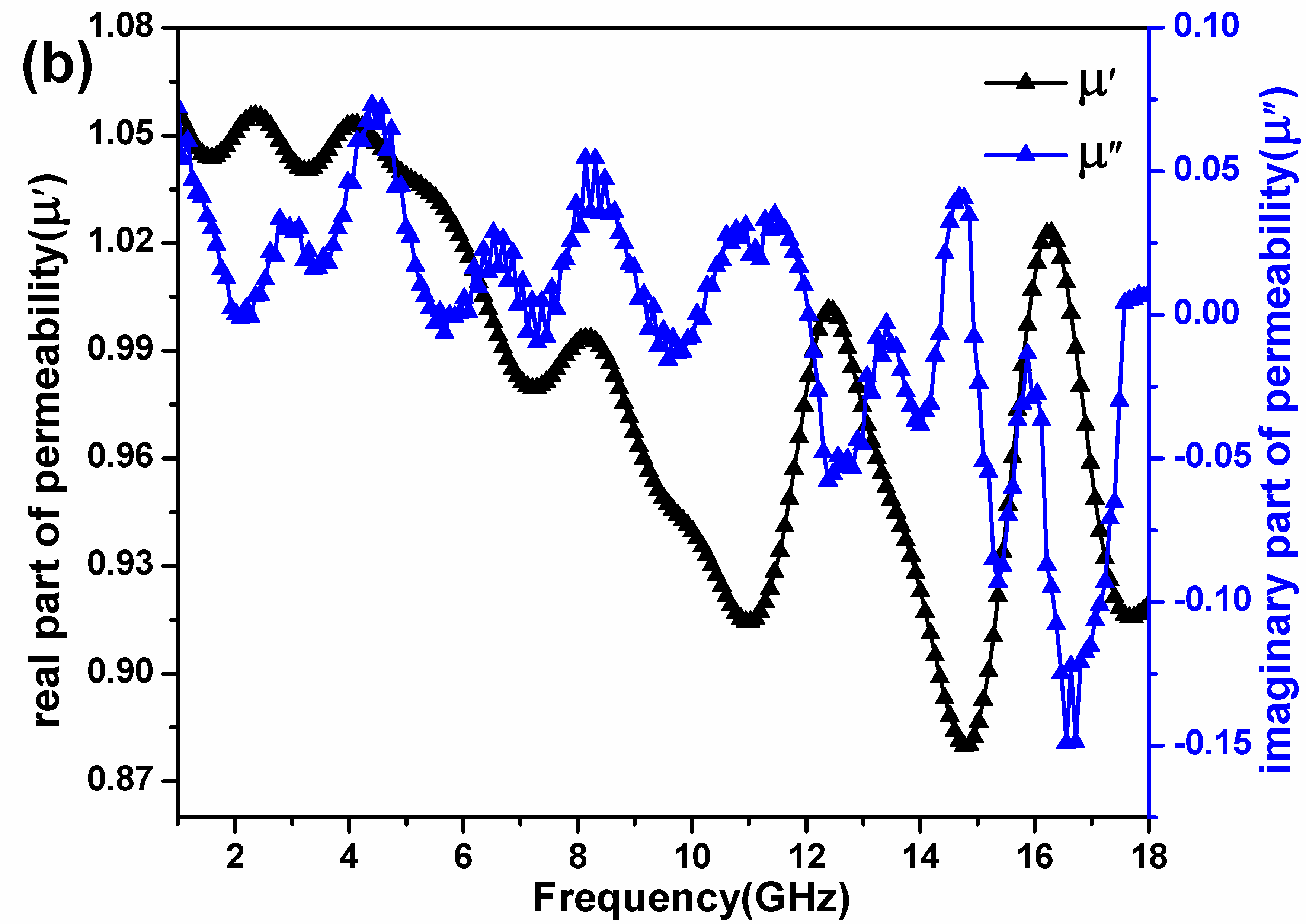


Fig.S3 Relative complex permittivity (a), relative complex permeability (b), of paraffin composites filled with 33.3 wt% S_0_


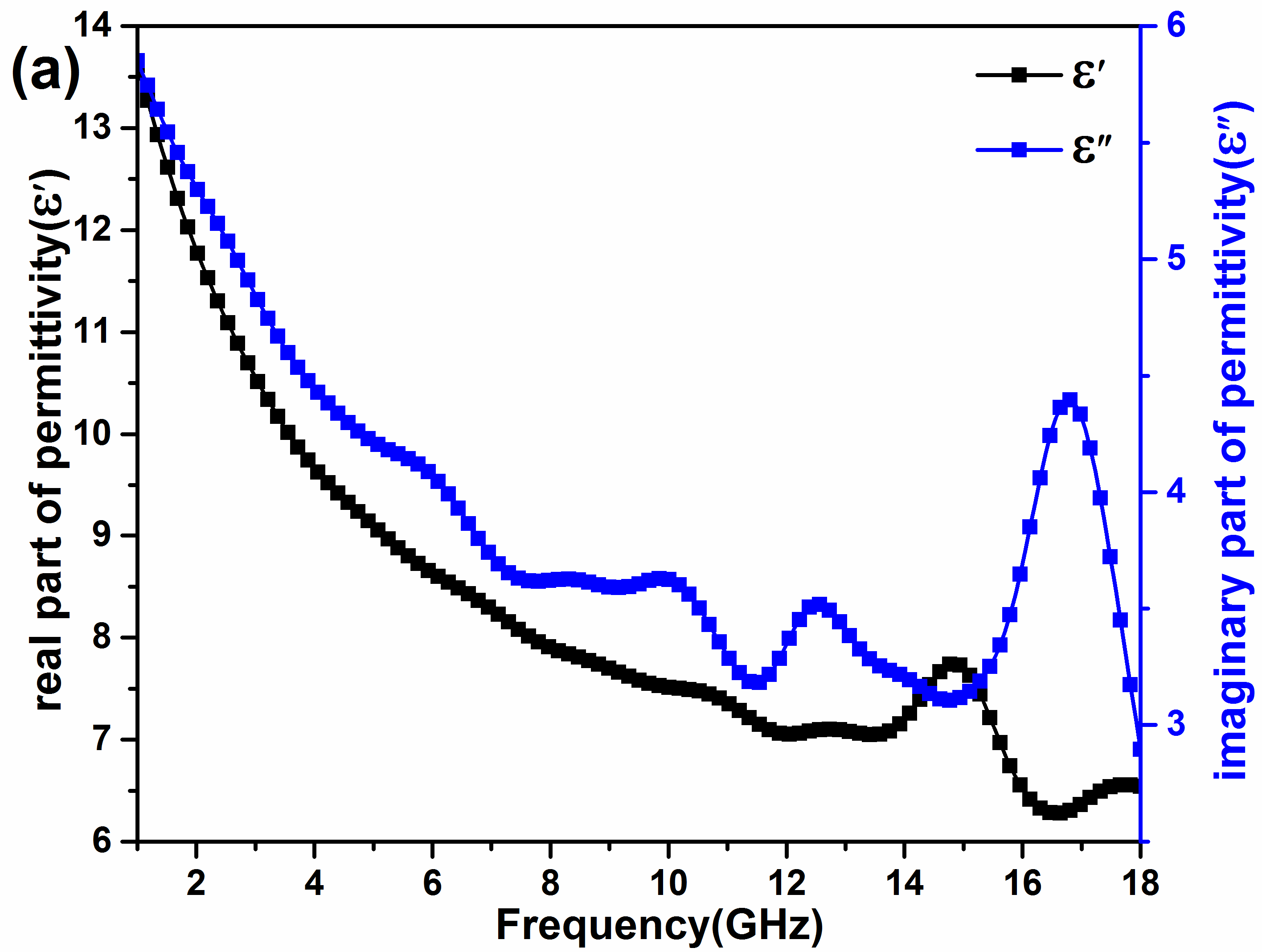

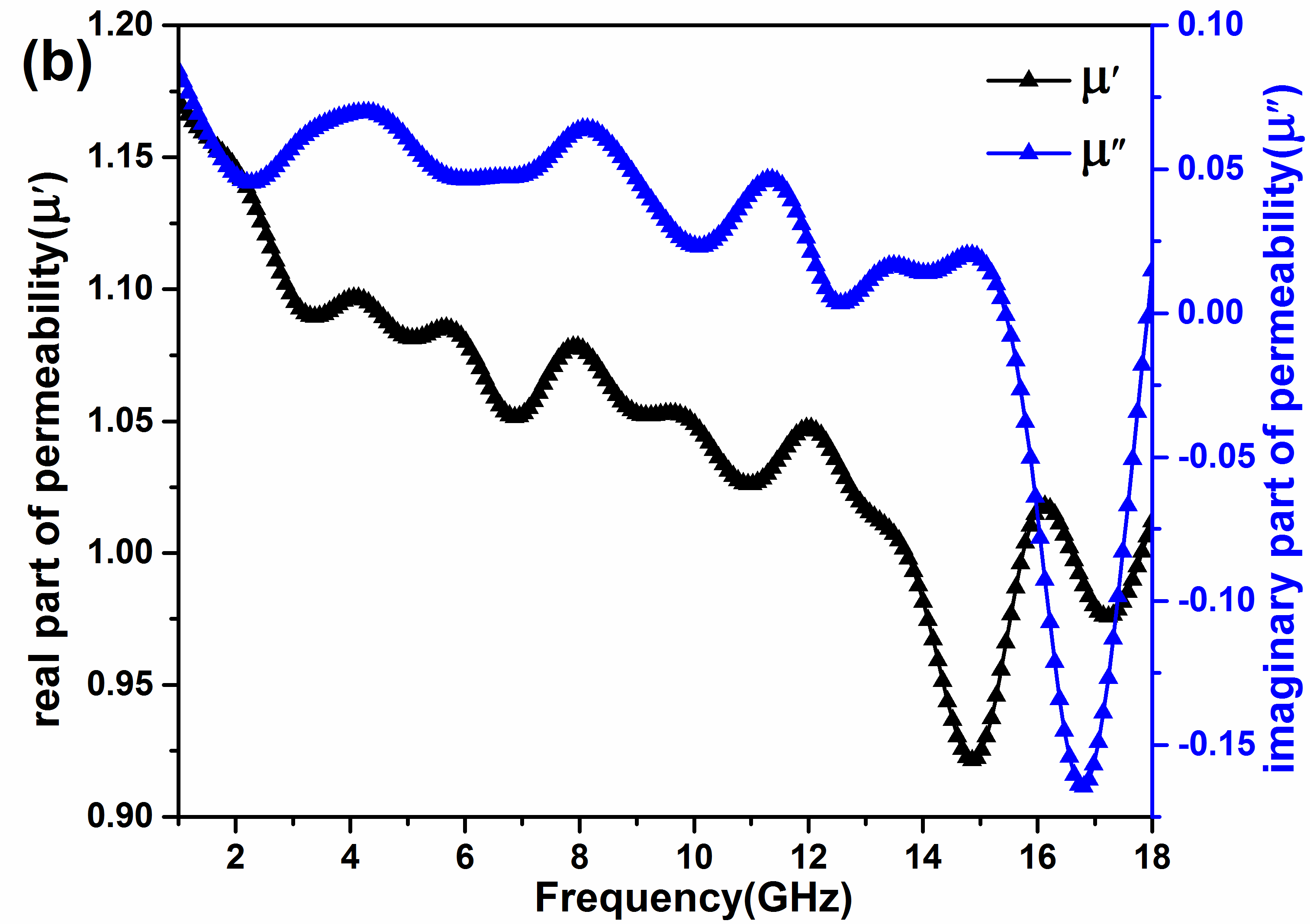


Fig.S4 Relative complex permittivity (a), relative complex permeability (b), of paraffin composites filled with 33.3 wt% S_0.5_


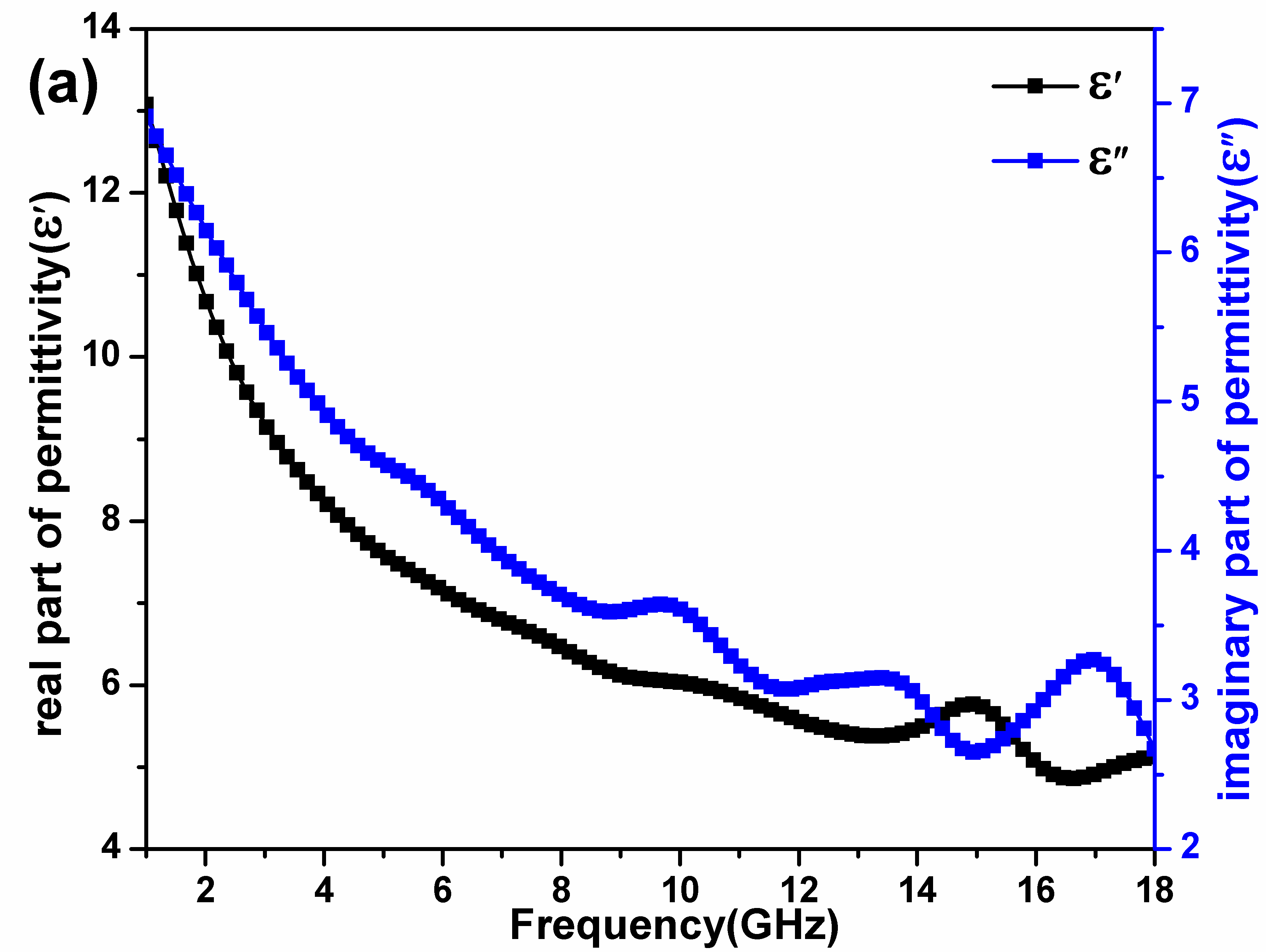

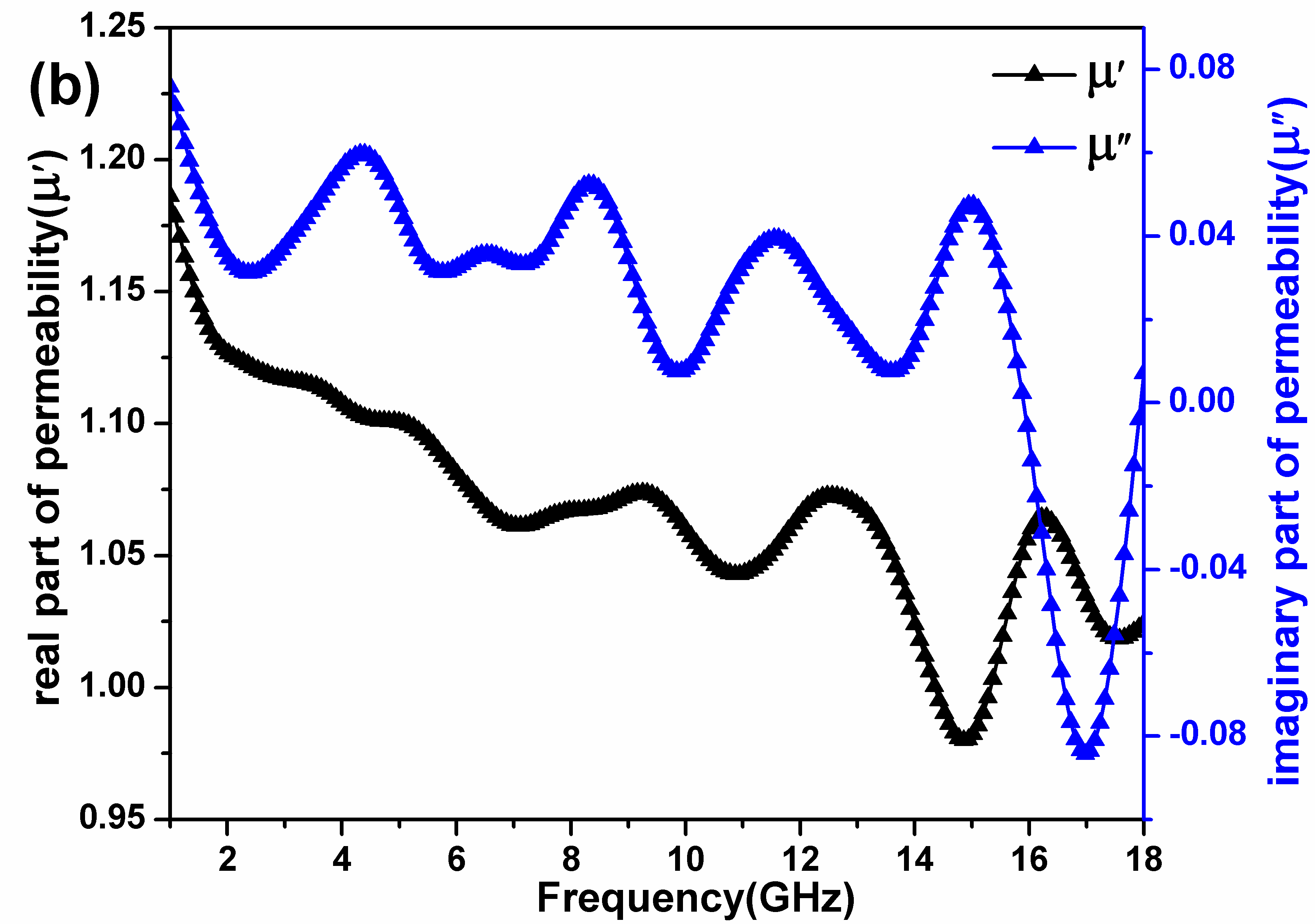


Fig.S5 Relative complex permittivity (a), relative complex permeability (b), of paraffin composites filled with 33.3 wt% S_1.0_


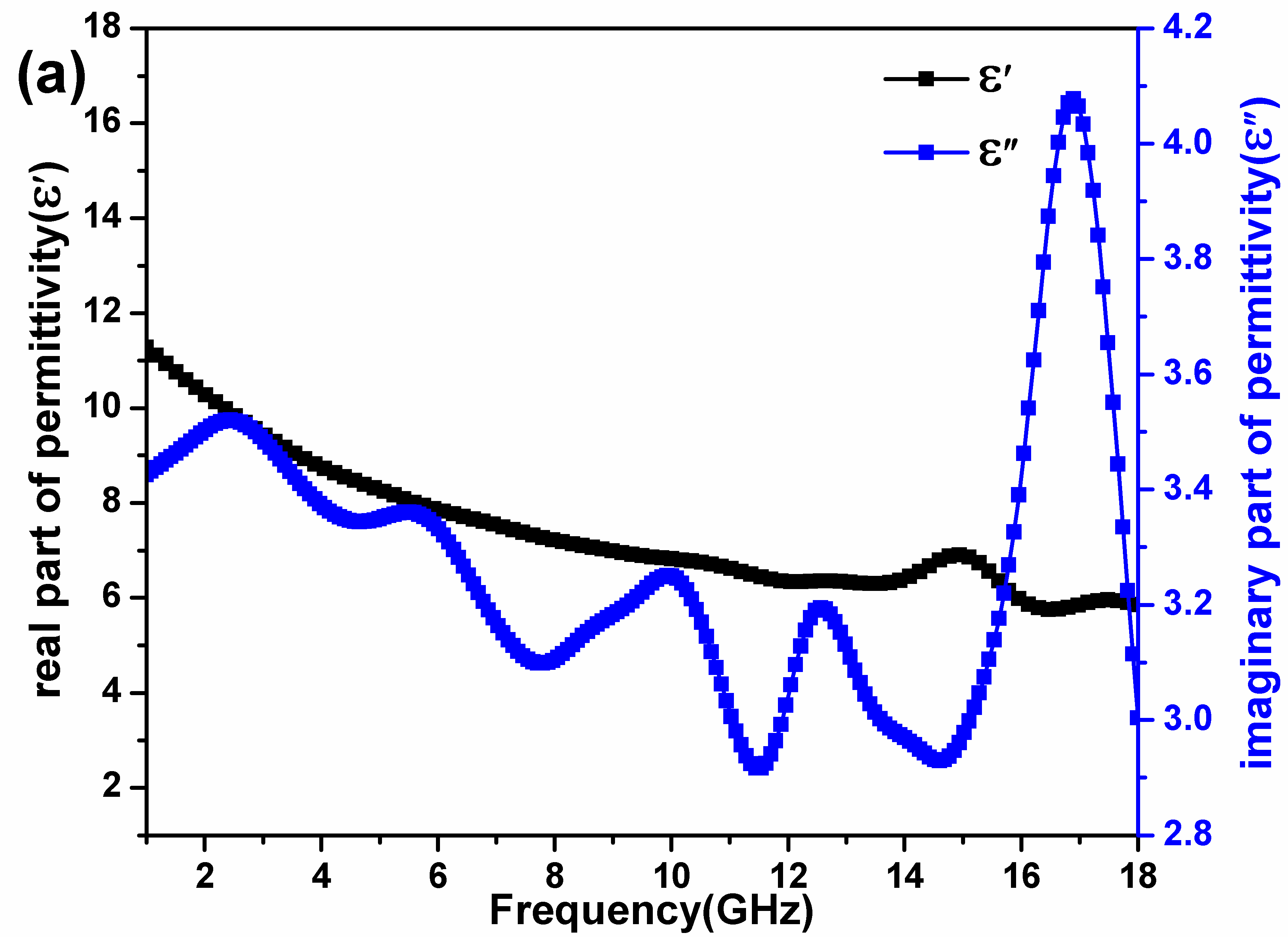

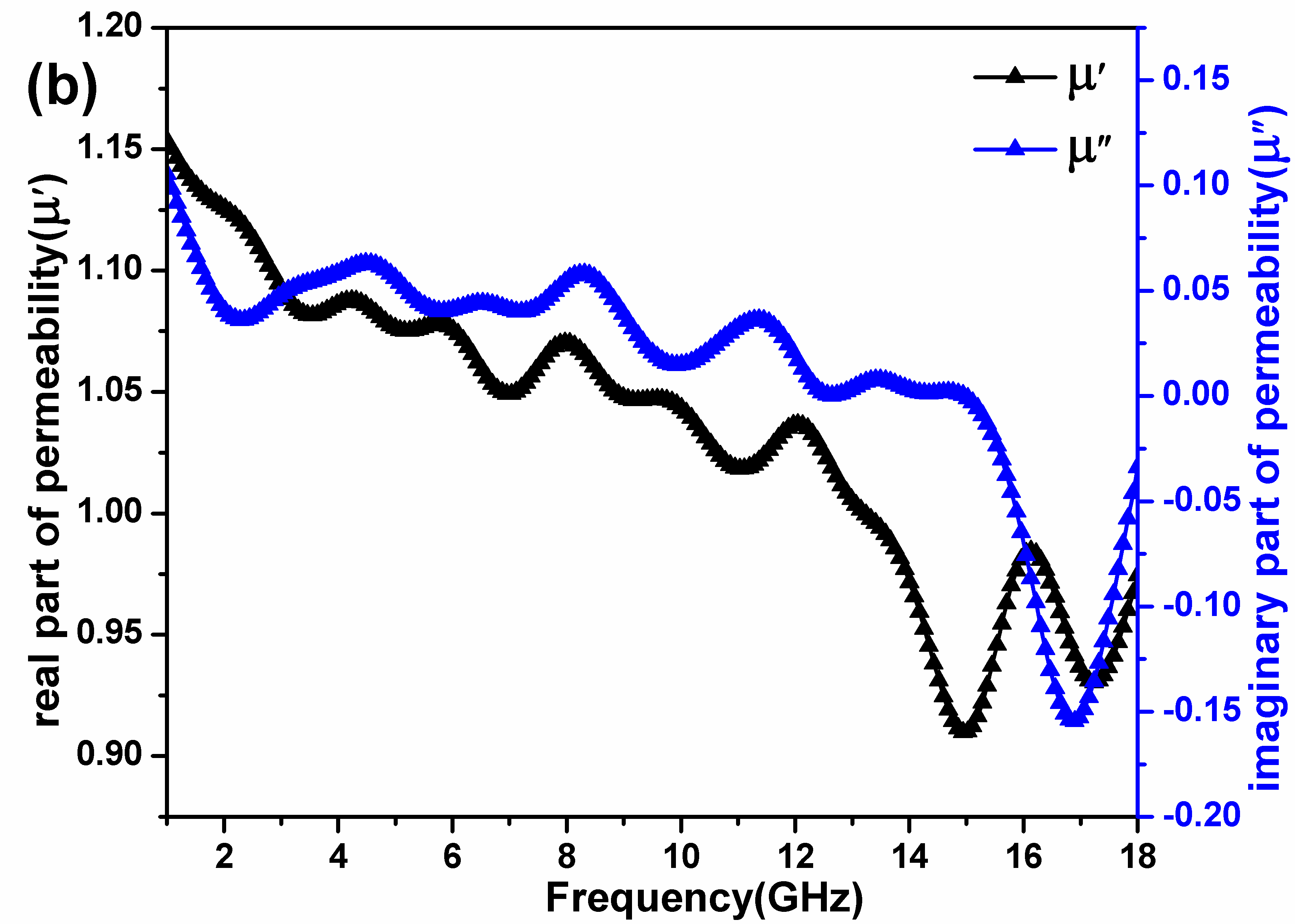


Fig.S6 Relative complex permittivity (a), relative complex permeability (b), of paraffin composites filled with 33.3 wt% S_2.0_


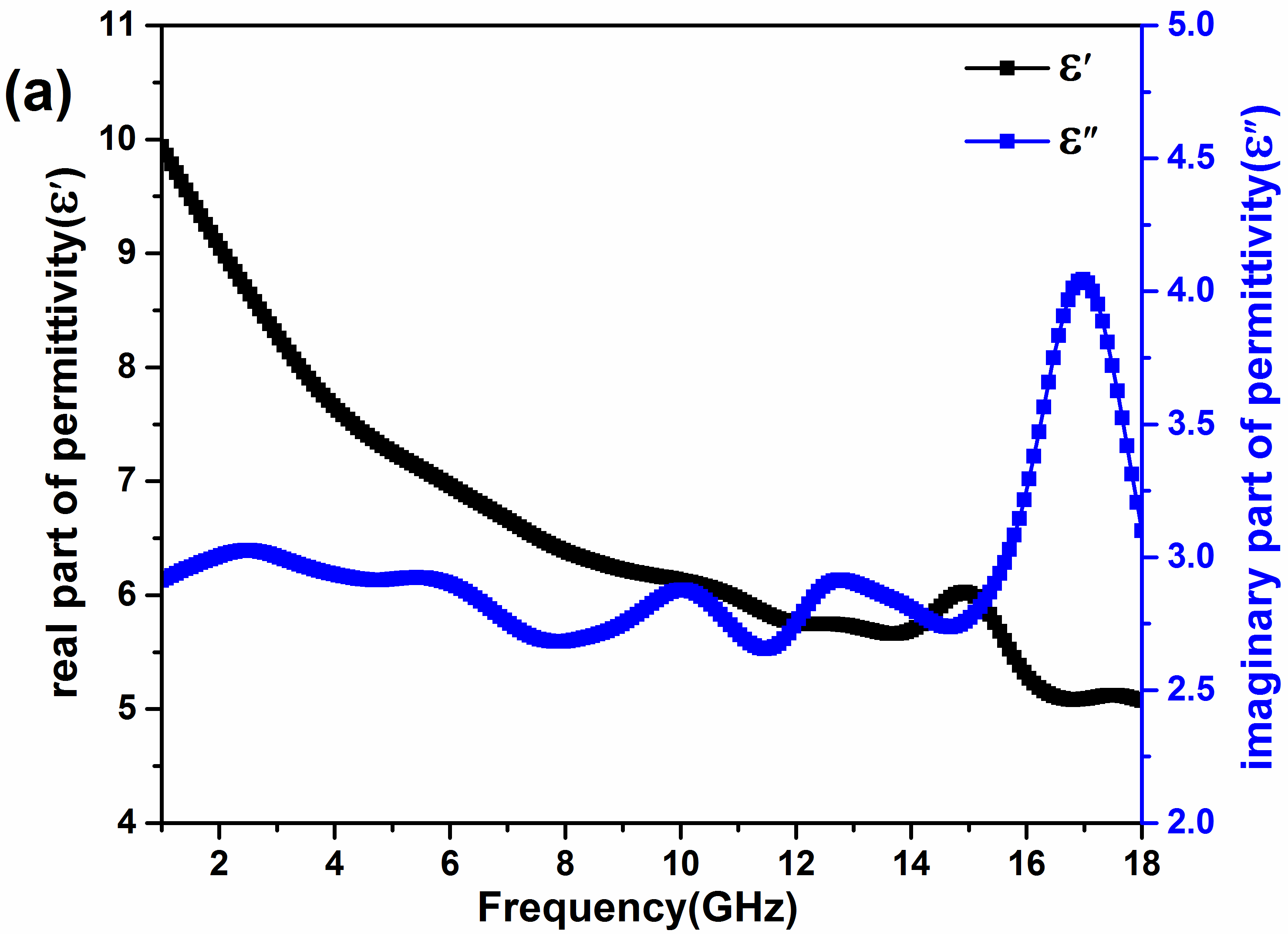

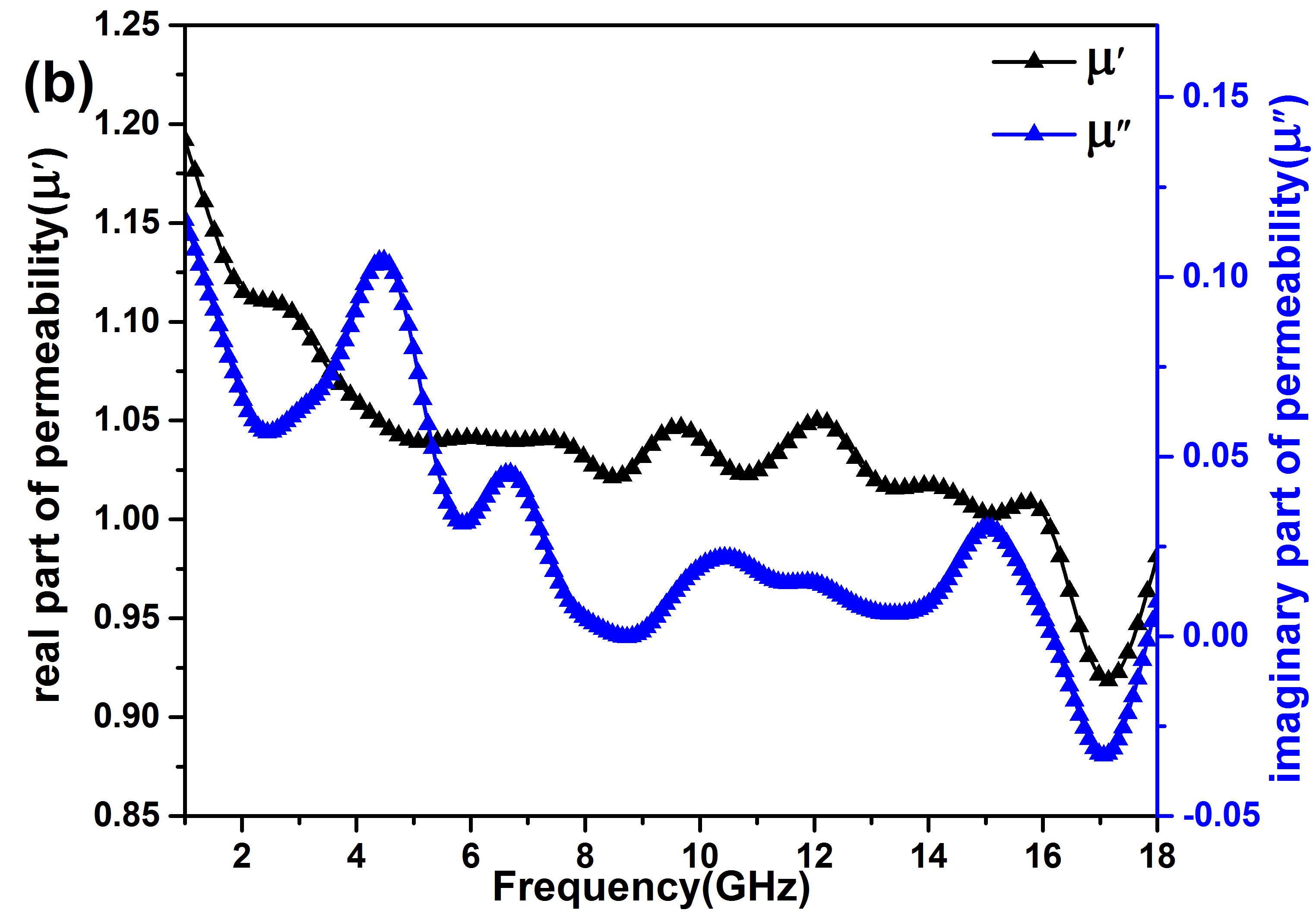


Fig.S7 Relative complex permittivity (a), relative complex permeability (b), of paraffin composites filled with 33.3 wt% S_2.5_


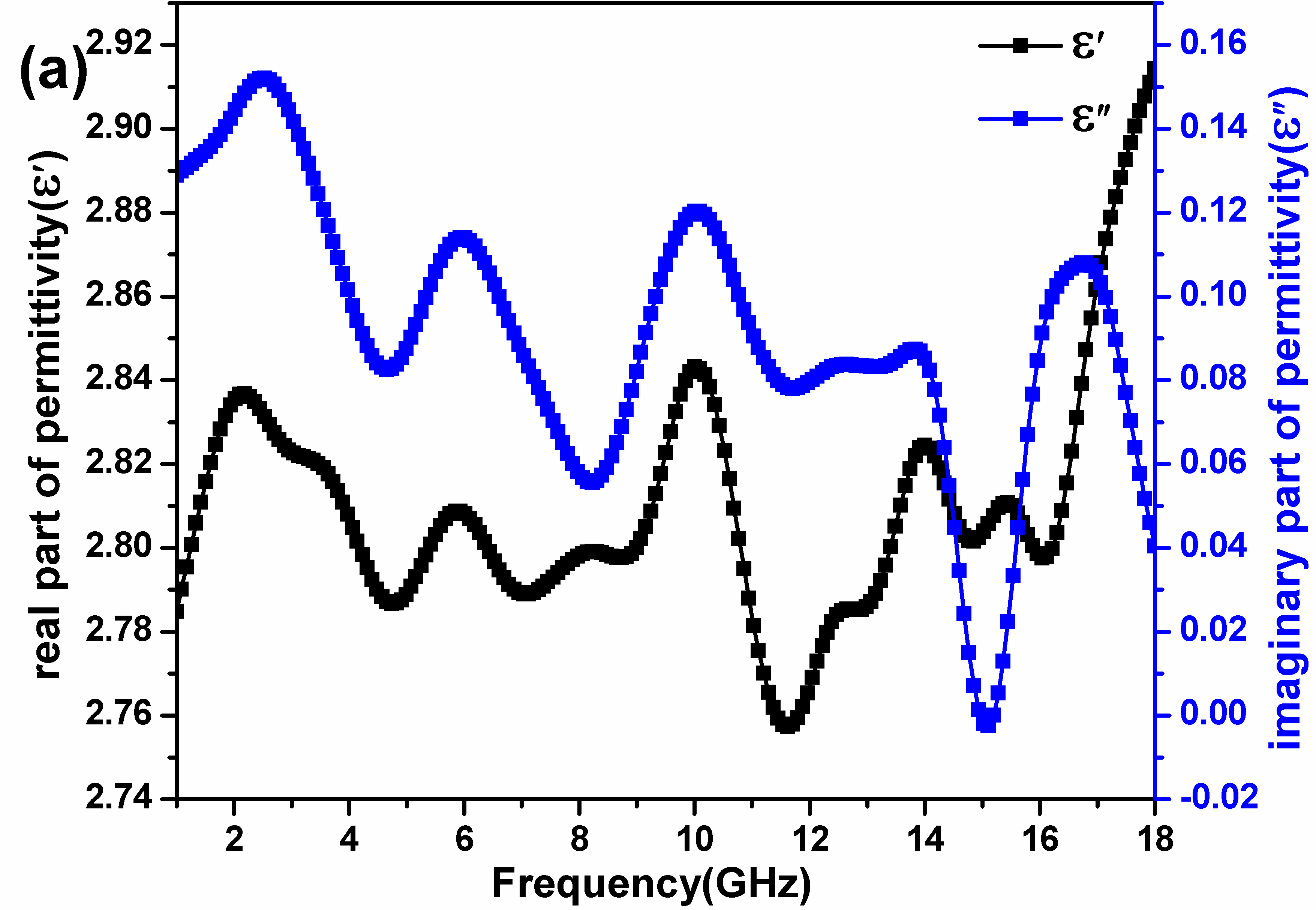

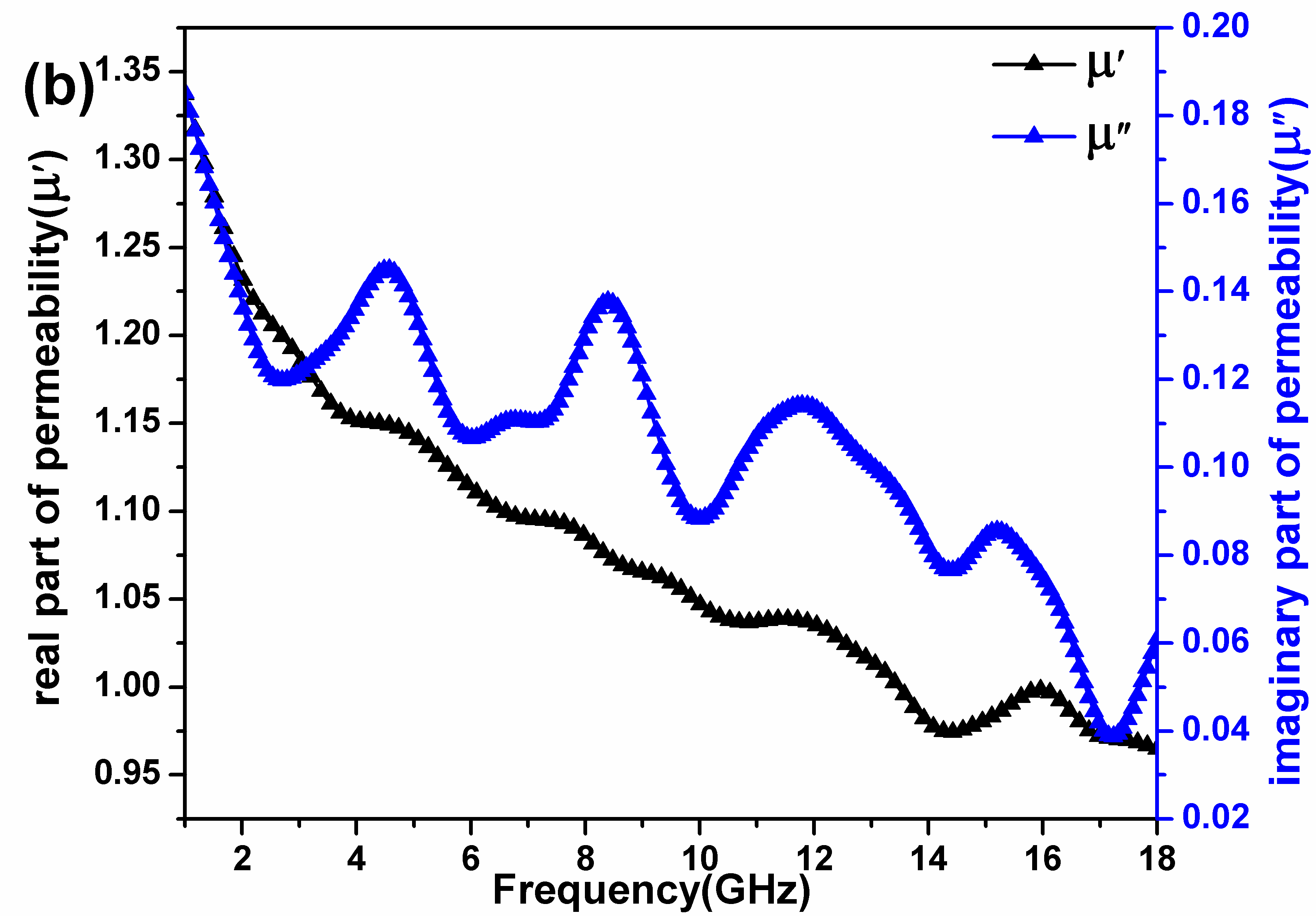


Fig.S8 Relative complex permittivity (a), relative complex permeability (b), of paraffin composites filled with 33.3 wt% S_p_
